# Continuity of care as experienced by mental health service users - a qualitative study

**DOI:** 10.1186/s12913-017-2719-9

**Published:** 2017-11-21

**Authors:** Eva Biringer, Miriam Hartveit, Bengt Sundfør, Torleif Ruud, Marit Borg

**Affiliations:** 1 Helse Fonna Local Health Authority, P.O. Box 2170, N-5504 Haugesund, Norway; 2 Regional Research Network on Mood Disorders (MoodNet), Haukeland University Hospital, Division of Mental Health, P.O. Box 1400, N-5021 Bergen, Norway; 3 Division of Mental Health Services, Akershus University Hospital, P.O. Box 1000, 1478 Lørenskog, Norway; 4 Institute of Clinical Medicine, University of Oslo, P.O. Box 1171, Blindern, N-0318 Oslo, Norway; 5 Faculty of Health Sciences, University College of Southeast Norway, Papirbredden, Drammen kunnskapspark, Grønland 58, 3045 Drammen, Norway

**Keywords:** Recovery, Mental health, Social welfare, Health care transition, Continuity of care, Patient experiences, Therapist change, Physician–patient relations, Information, Quality of health care

## Abstract

**Background:**

People who struggle with mental health problems can provide valuable insight into understanding and improving the coordination of mental health and welfare services. The aims of the study were to explore service users’ experiences and perceptions of continuity of care within and across services relevant to personal recovery, to elicit which dimensions of continuity of care are most essential to service users, and to generate ideas for improving service users’ experiences of continuity of care.

**Methods:**

In the context of a hermeneutic-phenomenological approach, ten service users at a community mental health centre were interviewed about their experiences of continuity of care in and across services. Eight of these were re-interviewed two years later. A collaborative research approach was adopted. Data were analysed by means of a data-driven stepwise approach in line with thematic analysis.

**Results:**

Following the analysis five themes representing experiences of continuity of care were developed. Each theme ranged from poor to good experiences of continuity of care: *Relationship* – from experiencing frequent setbacks and anxiety due to breaks in relationships, to feeling safe in an ongoing personal relationship; *Timeliness* – from experiencing frustrating waiting times with worsening of problems, to getting help when needed; *Mutuality* – from having a one-sided struggle, to a situation in which both professionals and service users take initiatives; *Choice* – from not having the opportunity to make practical arrangements within the context of one’s everyday life, to having an array of support options to choose from; *Knowledge* – from feeling confused and insecure because one does not know what is happening, to feeling safe because one is informed about what is going to happen. Participants provided a range of suggestions for improving experiences of continuity of care.

**Conclusions:**

A discrepancy between aspects of continuity that are essential for service users and their experiences of actual practice was revealed. The valid evidence generated in the present collaborative study therefore offers knowledge to policy makers, professionals and service users that may be of help in their future efforts in orienting primary care, mental health, addiction and welfare services towards recovery.

**Electronic supplementary material:**

The online version of this article (10.1186/s12913-017-2719-9) contains supplementary material, which is available to authorized users.

## Background

Continuity of care is considered by service users as well as professionals as an essential feature of high quality health care. However, there is no uniformity in existing definitions of the concept of continuity of care [1–5]. According to existing definitions based mainly on the perspective of health professionals, continuity of care is a multidimensional and hierarchical concept [1, 2, 4, 6]. It ranges from the basic availability of information about the service user’s past to a complex interpersonal relationship between the health professional and service user, characterised by trust and a sense of mutual responsibility. In a multidisciplinary review, Haggerty et al. (2003) conclude that three types of continuity exist in health care [6]: *Informational continuity*, where information is the common thread linking care from one provider to another and from one healthcare event to another. *Management continuity* is achieved when services are delivered in a complementary and timely manner, providing a sense of predictability and security in future care. *Relational continuity* represents ongoing personal relationships between the service user and one professional or a consistent team of professionals. Two elements are intrinsic within these three dimensions of continuity. The first element is care of an *individual* service user, in which continuity is represented by how the individual service user experiences integration of services and coordination. The second element is *longitudinality*, in which time distinguishes continuity from other attributes such as the quality of interpersonal communication during a single encounter.

The service user perspective is a valid perspective in quality of care, besides the professional and management perspectives [7]. Unfortunately, more recent empirical investigations point to differences between conceptualisations of continuity of care generated within the ‘professional paradigm’ [8] and studies focusing on the views and experiences of service users [2, 9–14]. It has recently been argued that current conceptualisations of continuity of care do not adequately account for the range and emphasis of definitions highlighted by either mental health service users or health care professionals [14–16]. The poor clarity and questionable validity of current conceptualisations of continuity of care for the service user perspective can be linked to a lack of service user involvement. However, studies on continuity of care that have been undertaken from the perspective of service users are hard to find [17].

Service users in mental health frequently have needs that are comprehensive and related to health, psychosocial and economic aspects [18, 19]. Continuity of care is a prerequisite for the provision of high quality care to meet service users’ needs. Earlier studies have shown that service users value easy and timely access to services [16, 20–23] and flexible and responsive care [9, 21, 24, 25]. Further, service users value care planning and coordinated transitions [3, 9, 21, 22, 26–28], and sufficient information and transfer of information [21–23, 27, 29, 30]. However, there is still a need for more research on the questions of what service users regard as continuity of care and how, in their view, continuity of care could be improved. We therefore set out to perform the present user-involved collaborative study to explore the experiences and perceptions of continuity of care from the service user perspective.

‘Recovery’ in and from severe mental health issues is a multifaceted personal and social process [31, 32]. Therefore, experiences with a broad range of services supporting recovery were included in the present study, including mental health specialist services, primary care services and employment and social welfare services. The study aimed at exploring service users’ experiences of continuity of care within and among these services. The following research questions were developed: Which dimensions of continuity of care are most essential for service users with mental health problems? How do service users experience continuity within these dimensions? How, according to service users, could continuity in and across services relevant to mental health recovery be improved?

## Methods

### Design

The study is a qualitative study. As the study aimed to explore how individuals with mental health problems experience health care and welfare services in the context of their current life and situation, a hermeneutic-phenomenological approach was chosen [33, 34]. A hermeneutic-phenomenological approach is suited for in-depth explorations of phenomena and questions of personal experiencing and understanding. Within a user-involved collaborative framework, ten service users were interviewed about their experiences with health and welfare services at the start of their mental health treatment (T0). Eight of these participants were available for follow-up interview two years (27 to 30 months) later (T1). A long follow-up period was chosen as personal recovery is typically understood as a long-lasting process, and a longer period would allow participants to gather more experience with using health and welfare services.

### Setting

**Fig. 1:**
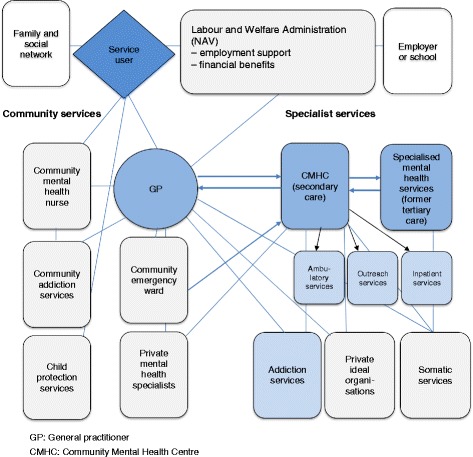
Study context: Norwegian health and welfare services relevant to mental health recovery Bold arrows indicate the commonly used paths of transferal of responsibility for providing care to service users. Narrow lines represent the main communication paths across services

In Norway, public mental health care services are organised along a continuum through which service users are expected to progress in a sequential manner (bold arrows in Fig. 1). Within the framework constituted by the community mental health services, specialised mental health services, employment and welfare services, and a few private institutions, a wide range of approaches and interventions relating to mental health problems are available. General practitioners (GPs) typically refer service users to a specialist mental health service. Most often patients are referred to a community mental health centre (CMHC, i.e. secondary care), for outpatient or inpatient treatment. Patients may also be referred directly to a psychiatric hospital (i.e. tertiary care). Community mental health nurses or mental health community teams are frequently involved in follow-up and rehabilitation. The most important institution besides the public primary and specialist care services is the Norwegian Labour and Welfare Administration (‘NAV’). The Labour and Welfare Administration is organised at the state level as a body separated from health care, but with extensions into the communities. The Labour and Welfare Administration is responsible for pensions, unemployment- and sickness benefits, qualification programmes and employment schemes, and it offers temporary financial assistance, temporal accommodation, financial advice and debt councelling. As mental health recovery happens within the context of material and financial safety [35–37] and meaningful activity (such as a job) [38, 39], including these employment and welfare services thus is particularly relevant from a recovery perspective.

### Participants

Participants for the study were recruited from a typical CMHC in Norway providing specialist mental health services to four municipalities (34,000 inhabitants). The CMHC hads outpatient clinics, outreach services, and two inpatient units for adults in addition to child and adolescent mental health services. Service users who were referred to the CMHC were recruited by their therapist at the start of their contact there in 2011 and 2012 (T0). Ten participants were recruited, ranging from 18 to 54 years of age (the mean age was 33). Four were women and six were men. At the follow-up interview (T1) approximately two years after the first interview, one out of the ten participants could not be traced and one declined the invitation to participate. The present study is therefore based on ten T0 interviews and eight follow-up interviews (T1). Both interviews (T0, T1) from each participant were included in the analysis. All participants provided written informed consent to participate. Table 1 describes each participant’s mental health problems, life situation, and use of services relevant to recovery.

**Table 1:** Participants’ mental health problems, life situation and use of services relevant to recovery^a^

P	Mental health problem (T0)	Life situation (T0 and T1)	Use of health, social or employment services (T0 and T1)
1	Chronic bodily pains	Married	First time outpatient in mental health services (T0)
	Fatigue	Part-time job	GP
			Work assessment allowance from the Labour and Welfare Administration Has requested disability pension from the Labour and Welfare Administration (T1)
2	Social anxiety	Co-habiting	Previously used child and adolescent psychiatric services and child protection services
	Periods of extensive cannabis use	Not employed	First time mental health ambulatory team
			GP
			Somatic specialist services
			Financial support from the Labour and Welfare Administration
3	Depression	Single	Previously used child and adolescent psychiatric services
	Anxiety	Completed secondary education	First time outpatient in mental health services (T0)
		Not employed	GP
4	Bipolar type II	Married	Private psychiatrist
	Periods of high alcohol intake	Full-time job	First time outpatient in mental health services (T0)
			GP
5	Depression	Married	First time outpatient in mental health services (T0)
		Full-time job	GP
6	Substance and alcohol abuse	Living with parent	Previously used mental health and addiction services
	Social anxiety	Not employed	Several previous hospitalisations in mental health services
	Depression	Working at the church charity centre (T1)	Ambulatory mental health team
			GP
			Somatic specialist services
			Financial support from the Labour and Welfare Administration
7^b^	Depression	Living with parents	First time ambulatory mental health team (T0)
	Anxiety	Full-time job	GP
	Previous periods of daily cannabis use	Previously unemployed	
8	Psychotic episodes	Living alone	Inpatient services (T0)
		Higher education partly completed	Several past hospitalisations in mental health services
		Not employed (T0)	GP
		Part time employment (T1)	Financial support from the Labour and Welfare Administration
9^b^	Alcohol abuse	Living alone	Many previous hospitalisations in mental health services
	Social anxiety	Previous full-time job	Regular visits by community mental health nurse
	Depression	Not employed	
10	Depression	Single	Outpatient services (T0)
	Delusions	Living with parents (T0)	One previous hospitalisation in mental health services
		Has his own apartment (T1)	GP
		Unemployed (T0)	Somatic specialist services
		Full-time job (T1)	After T0 job course and supported employment via the Labour and Welfare Administration

^a^Information provided regards both time points (T0, T1) if not otherwise specified

^b^First interview only

T0: First interview, *T1* Second interview two years later, *GP* General practitioner

### User-involved collaborative research

In order to ensure the validity and relevance of the research questions, analysis, conclusions and dissemination for the service user perspective, the present study employs a user-involved collaborative approach [40]. Involving service users in research is appropriate when exploring the service user perspective, which is a valid perspective on quality of care [7]. The semi-structured interview guide was developed in collaboration with the ‘expert-by-experience panel’ of MoodNet, a regional research network in western Norway [40]. The resulting semi-structured interview guide was then piloted by the help of 12 panel members with experience as service users in mental health care. A co-researcher (BS) with many years of experience in using mental health and welfare services took an active part in interviews, data analysis, and dissemination of results.

### Qualitative interviews

The semi-structured in-depth interviews were conducted by BS and EB at the CMHC and each individual interview lasted approximately one hour at each time point (T0, T1), respectively. The participants were invited to choose the interview setting they preferred, such as their home, a café, the CMCH or another public place. All of the participants choose to be interviewed at the CMHC. At the start of the interview, participants were introduced to the interviewers and informed that the aim of the study was to explore the participants’ experiences with health care services and mental health recovery. The participants were informed about BS’ background as mental health service user and EB was introduced as a ‘researcher’. The questions included in the semi-structured interview guide for the first (T0) and second (T1) interview can be found in Additional file 1. A central element in phenomenology is that lived experience provides meaning to every individual’s interpretation and understanding of a phenomenon [33, 34]. Therefore, in order to facilitate the exploration of the idiosyncratic experiences and understandings of each participant and his everyday life contexts, the semi-structured interview guide for the in-depth interviews included themes and questions that were open-ended in nature. The interview guide invited the participants to reflect on themes such as personal preferences and treatment goals, experiences with help-seeking and access to care, and experiences in mental health or social care, including experiences of being transferred to or discharged from health care units, changing therapist or contact person, transferal of information between involved parties, and information about treatment and treatment plans. Questions in sections B), F) (both at T0) and E), G) and H) (the latter three at T1) in the semi-structured interview guide concerned the exploration of participants’ experiences with aspects of continuity of care. Examples of questions from the interview guide include the following: ‘How –in your experience- did the cooperation between the CMHC, your general practitioner, and other contacts in health care or the Labour and Welfare Administration function?’, ‘Are there ways in which these parties could have collaborated better?’, ‘If these parties had worked better together, how, in your opinion, could this have affected your situation?’. Follow-up questions were posed in which the participant was asked to elaborate on his answer, for instance by describing how he experienced a change of therapist or contact person, and which emotional and practical consequences these changes brought upon him. In case of emotional distress after the interview the interviewer would assist the service user to contact his therapist (however, none of the participants showed signs of such distress at the end of the interview). Interviews were audiotaped and transcribed verbatim.

### Data analysis

Acknowledging that the researchers’ own involvement and prior understanding may impact on which knowledge is acquired, reflexivity was emphasised throughout the research process [34, 41]. Reflexivity was emphasised through reflexive collaboration and exchange of ideas with service users during the entire research process. Further, all researchers aimed at being aware of how their basic assumptions and values could affect the questions asked and conclusions drawn. A data-driven stepwise procedure in line with thematic analysis was used [42]. Data analysis proceeded as follows: EB read all material. Using NVivo 9, she systematically coded all text material and defined the preliminary themes. During two collaborative one-day workshops, BS and EB read, discussed, and agreed about a common understanding of the semantic and latent constructs underlying the material in the preliminary themes. No major differences in interpretation appeared among the researchers during these discussions. Based on the common understanding reached in the workshops, EB made the final categorisation of contents and drafted the manuscript based on notes from the workshops and original transcripts. Analysis was performed across the sample at each time point. For the eight participants who had participated at both interviews, data were analysed longitudinally with regard to intra-individual changes in insights and perceptions. To ensure the reliability of the findings, i.e. consistency of data, results and interpretations within the study, the results were compared with the original transcripts throughout the writing process. Further information about recruitment of participants and the methodological approach used in the study can be found in Biringer et al. (2015, 2016) [43, 44]. The study was approved by the Norwegian Social Science Data Service (ref. no. 22920/2). Additional file 2 shows the completed 32-item COnsolidated criteria for Reporting Qualitative research (COREQ) checklist for the study.

## Results

At the first interview (T0), all participants were suffering from severe mental health problems (Table 1). At the follow-up interview two years later (T1), participants were either partly or completely recovered. Five of the eight participants who were re-interviewed did no longer receive treatment at the CMHC. One participant who functioned well saw a private psychiatrist at irregular intervals. Two participants still had regular contact with services at the CMHC. Six participants had experienced that professional help played a role in their recovery [44]. The participants described experiences with mental health and addiction specialist services, primary health care, the Labour and Welfare Administration, the Child Protection Service, somatic institutions, and private therapists or institutions (see Table 1). Five main themes reflecting participants’ experiences of aspects of continuity of care and their emotional and behavioural reactions to these experiences are presented in Table 2. The five themes were as follows:

- *Relationship* – from experiencing frequent setbacks and anxiety due to breaks in relationships, to feeling safe in an on-going personal relationship
- *Timeliness* – from experiencing frustrating waiting times with worsening of problems, to getting help when needed
- *Mutuality* – from having a one-sided struggle, to a situation in which both professionals and service users take initiatives
- *Choice* – from not having the opportunity to make practical arrangements within the context of one’s everyday life, to having an array of support options to choose from
- *Knowledge* – from feeling confused and insecure because one does not know what is happening, to feeling safe because one is informed about what is going to happen

**Table 2:** Service users’ experience of continuity. Themes represent continuums from good to poor continuity

Theme	Good continuity		Poor continuity	
	Description	Subjective experience	Description	Subjective experience
Relationship	Trusting relationship with one or a few professional helpers over time	Mutual knowledge and respect Feelings of trust and safety Perceiving support as helpful	Frequent breaks with therapist or contact person(s)	Having to tell your personal story again and again is frustrating Having to relate to new persons provokes anxiety Feeling rejected Getting the impression that the professional helpers do not care Setback in terms of diagnostic evaluation and treatment
Timeliness	Help when needed Not having to wait	Feelings of relief Avoid negative consequences of waiting too long	Being kept waiting Not knowing what is going to happen	Worrying about problems and upcoming contact with services Experiencing challenges with managing mental health and related problems Suffering and worsening of problems Risk of suicide
Mutuality	All involved parties take initiatives Having an opportunity for contact whenever something comes up Having a say in decisions	Feeling that the professional helper is reliable and cares about you Feeling that you have a say in decisions	Always being the one who has to take the initiative in order to make things happen	Feelings of frustration and indifference, feeling that you have to ‘fight’ the system Feeling ignored because of professional helpers who do not get in touch
Choice	Having the opportunity to choose among an array of options regarding where to be treated and what kind of support to get Having the opportunity to influence decisions Having the possibility of increasing personal continuity by making individual choices suited to your situation and context	Feeling that the situation is created according to your needs, both regarding treatment and practical aspects	Having no choice regarding decisions about where, when and how to get help No possibility of influencing decisions about contact persons, treatment and support Following the rules made by the system, for instance when being transferred from one service to another	Feeling ignored Feelings of indifference or opposition towards professional helpers, treatment and the system Starting to ignore the system and its rules
Knowledge	Knowing about evaluations and future plans Getting information about scheduled meetings and support interventions well ahead of time Knowing who is communicating about you, and how and why	Understanding what is happening and what is going to happen Feeling more secure and being more secure Experiencing predictability in practical terms	Not being informed about what is happening, and why and how Not knowing how or whether the involved parties communicate about you or your situation	Feelings of confusion, distress and insecurity Feelings of tiredness and indifference

**Fig. 2:**
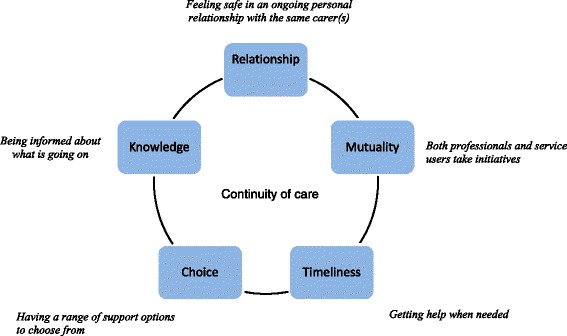
Dimensions of service users’ experiences of continuity of health and social care

Through the analysis, the participants’ experiences of continuity of care were found to represent continuums within each of these five main themes. At one end of each continuum we found experiences of good continuity, i.e. experiences that participants valued and described as useful (see Fig. 2). The other end represented experiences of poor continuity. Some degree of overlap existed between themes (Table 2). A final theme included participants’ ideas and suggestions for how experienced continuity of care could be improved (Table 3).

**Table 3:** Service users’ suggestions for how to improve experiences of continuity

Suggestion	Example	Relevant theme
Talk to each other	Talk to the other professionals involved about service users and their needs and care plans	Knowledge
Attend collaborative meetings	Contact persons in different services, i.e. general practitioner and contact persons in health or welfare services, should meet regularly and exchange information about and with service users about their situations and planned interventions	Knowledge Mutuality
Do not change contact person	Keep the same contact person over time, do not change contact person or therapist	Relationship
Inform service users in advance about changes in contact persons	Inform service users before changing contact persons. Failure to do so makes it seem like you do not care about them	Knowledge Relationship
Show service users that you care about their situations	Ask service users how they are doing, in addition to asking what you can do for them	Relationship
Take peoples’ anxiety into consideration	Take into consideration the fact that many service users suffer from anxiety in social situations and in situations where they have to deal with professional helpers	Relationship
Do not expect service users to act perfectly	Service users may have a feeling that the system demands they act perfectly in order to deserve help. Not feeling this demand would make it easier for service users to admit it when they are wrong	Relationship Mutuality
Work quicker	Do not take such a long time, for instance with making decisions that are important for the service users’ recovery or financial situation. Give the service users information swiftly	Timeliness Knowledge
Provide information about (planned) evaluations, treatments and support	Provide information ahead of planned treatment and care	Knowledge
Convey the same messages	Convey the same messages as the other professionals involved. Different information creates confusion	Knowledge
Make use of the waiting time	Offer an opportunity for someone to talk to during waiting time prior to treatment or interventions. During the waiting time, you could provide the service users with information about specific therapies or support interventions and about what is going to take place during the first meeting	Timeliness Knowledge
Do not be square	Do not follow rules systematically if the rules create impractical or paradoxical situations for the service users	Choice Mutuality
Be open to contact, also between scheduled appointments or across service boundaries	Be open for contact, for instance by being available by telephone between scheduled appointments, or after the therapy has ended	Mutuality Choice
Provide the person with follow-up over time	Schedule a follow-up appointment with the service users after their treatment or contact has ended, for instance some months ahead. Such an appointment gives service users a feeling of security, and they get an opportunity to discuss their problems and repeat what they have learned about how to deal with the problems	Relationship
Include family in information and contact	Invite next-of-kin to meetings and share relevant information with them in other ways	Knowledge Relationship
Provide general information about mental health problems, available services and treatments	Make general information available, for instance on the internet pages of the municipality, in media or schools. Include telephone numbers and information about where to find help	Knowledge

### Participants’ experiences of continuity of care

The participants’ experiences of collaboration and continuity between the various professional helpers and services involved varied a lot. Some participants were satisfied with the care and support they had received, even if they had experienced changes in contact person and had not always been given the necessary information about treatment and future plans. Several participants experienced multiple discontinuities in their contact with health and welfare services. These discontinuities frequently happened as they were transferred from one service to another, for instance from a psychiatric hospital to a CMHC, or from an inpatient unit to an outpatient unit at the same CMHC. Sometimes the disruptions in the personal relationships arose because the therapist or primary contact person went on leave or left his job. Some missed information about available services, support options and planned treatments or interventions well ahead in time, and some found the information they received confusing. There was little evidence of intra-individual changes in perceptions, insights and emotions with regard to experiences of continuity of care from T0 to T1. Below, the five themes, or continuums, representing dimensions of continuity of care that appeared as salient in the experience of the participants are described in more detail. The five themes are operationalised as continuums, ranging from experiences of good to poor continuity of care.

#### **Relationship** – from experiencing frequent setbacks and anxiety due to breaks in relationships, to feeling safe in an ongoing personal relationship.

The participants valued ongoing personal relationships with the same carer or contact person. Changes in carer or contact person were experienced as setbacks in treatment. These changes sometimes gave rise to feelings of anxiety, frustration, and a sense of being rejected. Some of the participants said that they had had enough of repeatedly having to tell their personal stories to new therapists or counsellors. One participant, a man with severe alcohol and drug problems who had been in contact with mental health and social care services since childhood, described his experience as follows:

‘It’s always the same. I have to tell my whole life story again and again. Even though they have the records, they still want to hear it. No, it’s happened too many times […].

It’s easier just to deal with one person. That’s so much better than being thrown around backwards and forwards between different social workers.’ (P6 (T1)).

Some participants described the change of therapist or contact person as a setback in their struggle towards recovery, because the new professional repeated tests and evaluations that had already been performed. For example, a woman who had to change GP several times described that:

‘What happens is that I begin from the beginning again, you know, because you've got to go through the whole process again, because they have to do their tests and their examinations to check that what the last doctor ended up with was right.’ (P1 (T1)).

Others experienced feelings of being ignored when they had to change contact person. A young woman who had experienced many breaks with family and professional helpers in her childhood dreaded having to start a relationship with a new psychologist so much that she refused the therapist she was offered at an outpatient clinic. She had previously been under protection of the Child Protection Services. According to her, her anxiety and distrust in situations where she had to change therapist had arisen as a result of her past experiences of frequent changes of case workers in the Child Protection Services:

‘They can read, so they know, but they ask. And you have to say the same things you said before. And when you have struggled to come out with it once, then it’s even more difficult the second time, and the third time, and fourth... It just gets worse and worse [...] right till it comes so far that it doesn’t mean anything anymore .... That’s when I start to get scared ...’ (P2 (T0)).

At the second interview, two years later, she felt ignored as her contact person at the CMCH intended to end their contact. She feared she would become so mentally broken that she would no longer be able to take care of her own child.

One of the participants, who had had an addiction problem since a very young age, experienced the recent involvement of the local assertive community treatment team as a positive change. To him, the visit by the two same team members every fortnight represented a safe, stable and predictable contact. Another participant, who used to manage his bipolar disorder quite well by himself, found the stability and trust he needed in a private psychiatrist. The meetings with the therapist three or four times a year seemed to be a kind of security mechanism for the participant:

‘Sometimes I'm a little close to the edge. I can have an unhealthy relationship with alcohol and stuff, typically when I travel or when things aren't so good, or a little good or a little too good, then that can be a part of it too. There he [the therapist] is good, with a way to tell me that “now you have to have a break”.’ (P4 (T1)).

The participant seemed to respect this health professional. Whenever the psychiatrist, with whom he had a longstanding relationship, told him to take it easy with his unhealthy habits, the participant would listen and respond accordingly.

Several participants described their GPs as a stable, but somewhat distant, contact in their lives. Most participants had a GP, but mainly went to see him for somatic problems, and rather hesitated involving him in their mental health issues. Some expressed warm feelings and gratitude to their GP for what he had done for them in terms of taking their side and helpfully intervening by referring them to services they needed.

#### **Timeliness** – from experiencing frustrating waiting times with worsening of problems, to getting help when needed.

Getting professional help when needed and before further damage happened was important to the participants. However, all except one participant described that they had to wait for access to services. Most of the informants found the waiting time frustrating and challenging, experiencing both hardships in their everyday lives and fears about the upcoming treatment. Some, such as a young participant suffering from severe depression at T0, felt that they should have been helped earlier, because then they would not have ‘fallen so low’ (P10 (T1)). Another participant dreaded the potential consequences of not getting help early enough:

‘The waiting time in situations like this is the worst. I would say suicide risk would be the worst, and that the situation or the suffering can get worse is bad enough.’ (P4 (T1)).

If free to choose, all the participants would have preferred shorter waiting times.

#### **Mutuality** – from having a one-sided struggle, to a situation in which both professionals and service users take initiatives.

Participants experienced the relationship between themselves on one hand and the helping services on the other as a one-sided relationship. In their experience, it was most often they themselves who had to take responsibility and initiatives in their contact with the health care and welfare services in order to get the help and support they needed or were entitled to. The participants frequently suffered from lack of energy and feelings of exhaustion due to their mental health problems. To some, having to pursue their own cases in the professional system felt excessively demanding. One of the participants described her experience with the helping system, in this case the Labour and Welfare Administration, as a constant struggle:

‘I had a battle with them too. [...] …Things take time and are difficult. It's really confusing […] when one person says one thing and another something else, then you get a letter with a third thing, and you just sit there ...’ (P2 (T1)).

The woman got increasingly frustrated and exhausted from ‘fighting the system’, and showed an indifferent attitude, expressing that she ‘can’t be bothered’. To get the help she needed, she threatened the health professionals she was involved with to ‘go crazy!’ (P2 (T1)).

Professionals who did not keep appointments were regarded by participants as unreliable and left them with feelings of disappointment. For instance, one woman lost trust in her GP after he did not turn up for a scheduled collaboration meeting about her situation to which several parties had been invited. One woman was pregnant at T0. During her pregnancy she was followed closely by her GP and members of a mental health ambulatory team due to her habit of cannabis smoking. Although she no longer used cannabis, she experienced constant feelings of anxiety as she had to wait three months for a home-visit from the Child Protection Service after her child was born:

‘They called me two days after I gave birth just to let me know that they would start a case and I had to be ready for it. Then it took nearly three months before they called again […]. And they switched the case officer. It was a man who called me first. And then when I called, I was told that I had no case worker. And then of course almost three months passed and a lady called and said “Now I want to meet you” and “Can it be next week?”…[…]. It was hard. It was a constant anxious feeling of “now they come and now they come and now they come and now they come and when do they come and what happens?”’ (P2 (T1)).

While using mental health services, participants wished for the opportunity to contact their professional helpers between scheduled appointments in case something came up. Some were offered the possibility of calling the CMHC during its opening hours. However, not all participants perceived this offer as genuine, as professionals frequently did not answer or return calls. Professionals very rarely contacted the participants between scheduled appointments. The participants very much appreciated professionals who contacted them between visits or who made follow-up contacts after care had been terminated:

‘The last time I was here [at the CMHC] there was [psychiatrist’s name]. […] As he was finished here, he left for [a neighbouring country]. And suddenly my phone rang. I think it was 14 days after he finished and went home. Then he called me from [the neighbouring country] just to ask how things were going!’ (P9 (T0)).

#### **Choice** – from not having the opportunity of making practical arrangements within the context of one’s everyday life, to having an array of support options to choose from.

Several participants appreciated the opportunity they had been given to choose treatment type or place, as well as the opportunity to be involved in deciding when and how the contact with their therapist should happen. The choices they were given had allowed them to make arrangements that were convenient to them and allowed them to take family, social or geographical circumstances into account. For instance, the man quoted above, who had a severe drinking problem, was happy that he could have a say about where he was admitted:

‘That was just great. That he [the GP] would have me go to either [name of CMHC] or [name of another unit], but I said I was not interested in those, because I wanted to be home. So, I myself suggested the CMHC here.’ (P9 (T0)).

A young woman who had experienced several psychotic episodes in the past was also happy that she could choose where to be admitted. She made her GP refer her to a more local CMHC as she again started seeing the shades on the wall that tended to precede a psychotic episode. Being admitted to the more local, but less specialised, centre allowed her to see her child and family more often. She also knew that centre’s facilities and health professionals well from earlier stays, and they knew her and her personal background.

Several participants, but not all, experienced that their voices were heard regarding the question of when and how their psychological treatment by their therapist should end, or whether they should move to another unit. A young depressed man who was very eager to find himself a job was impressed after he experienced how ready his doctor and counselors from the Labour and Welfare Administration were to let him choose between various options:

‘They are totally superb because they concentrate on you as a person in relation to “What do you want? What do you wish to happen? What can we help you with?” [...] Everybody was at the meeting and just [said] “OK, now we’re here for you.” And you feel completely overwhelmed. Four people sitting around me and just talking about you [me] and [saying] “Yes, what can we fix for you?” and so on. So, it has been very good. I have only positive things to say about both “NAV” and the doctor and the cooperation there …’ (P10 (T1)).

At the first interview (T0) he had been severely ill. Two years later (T1) he said that he had got a regular job that suited him well after first being helped to join a supported employment programme administered by the Labour and Welfare Administration.

Despite the examples above, most participants perceived the boundaries between the various services as very clear and strict, and exceptions to these boundaries were rare. One young woman explained her negative experience as she was transferred from child and adolescent services to adult mental health services:

‘It’s pretty important to me to have one person – not ten different people. That’s because of my trust issue ... when you go from the child and adolescent psychiatric services to adult mental health services, then you have to switch regardless. And I don’t think that’s good at all, because it’s not unusual to struggle with not wanting new people. When you have first told your whole life story to one person, and told everything you don’t want to share with anyone, to suddenly be thrown in another room, just because you’re 18 …’ (P2 (T0)).

Participants sensed that they were not supposed to contact professional helpers from their previous service after they had been transferred to another unit or service. For instance, none of participants had experienced being offered an opportunity of contact or systematic follow-up after discharge from the inpatient unit or the outpatient clinic.

#### **Knowledge** – from feeling confused and insecure because one does not know what is happening, to feeling safe because one is informed about what is going to happen.

In the participants’ experience, not knowing what was going on and was going to happen next was very distressing and sometimes provoked anxiety. The young woman above had to seek support from the Labour and Welfare Administration because of a distressing financial situation due to her mental health problems. She experienced misunderstandings and confusion in her communication with employees there:

‘There’s poor communication from their side and from my side at times, but that’s because I get fed up when I ring to find out what’s happening and I get three different letters within a week with three different appointments. They don’t speak to each other, I get totally ...’ (P2 (T1)).

‘Don’t you have a set case worker?’ (BS).

‘No, yeah, I think I’ve changed two or three times within two years.’ (P2).

‘Why?’ (BS).

‘I don’t know. I don’t get to know. It just says a new name at the bottom of the letter.’

‘But do you feel that the person that takes over knows what was done by the previous person?’ (EB).

‘I’ve still not spoken to the one who took over the last time. I didn’t feel it last time, because that was when we should start and so I got one first, and then she suddenly didn’t have anything to do with jobseekers allowance, so then I had to have another.’

Another participant experienced the lack of information about upcoming meetings with his therapist at the inpatient ward as distressing. He wished these meetings to be scheduled in the preceding meeting. However, the information that his therapist expected seeing him in his office at the CMHC was usually given to him as late as the evening before or the same day as the meeting.

Several participants were unaware of whether or how information about themselves and their issues was exchanged between the involved parties:

‘The doctor though, she is aware of what I have done there [at the CMHC], and when I have been there, and when I quit and so on.’ (P3 (T1)).

‘How does she know that?’ (BS).

‘They must have contact, I guess.’ (P3).

‘Does she have internet contact with the community mental health centre or by letter, do you know?’ (EB).

‘I’m not quite sure of that actually, but she must have some internet contact or something, I imagine.’ (P3).

‘“Discharge letter”, have you heard about that?’ (EB).

‘No.’ (P3).

It appeared as if the way the Labour and Welfare Administration informed service users about their legal rights and opportunities was not particularly well tailored to the recipients’ ability to absorb the information. One woman who at the re-interview recently had applied for a disability pension due to her bodily pains said she had received many information letters from the Labour and Welfare Administration. She seemed, however, to be unable to take this information in:

‘How has it been with information regarding rights and opportunities within the welfare centre?’ (EB (T1)).

‘It’s not especially good, is it?’ (P1).

‘I’m asking you.’ (EB).

‘I haven’t got a clue.’ (P1).

### Participants’ suggestions for how to improve experiences of continuity of care

The final theme revealed through the analysis concerns the participants’ ideas and suggestions for how to improve experiences of continuity of care. The suggestions based on participants’ experiences and opinions are summarised in Table 3. In Table 3 each suggestion for improvement is linked with one or more of the five themes above reflecting participants’ experiences. Most of participants’ advice concerned their desire for ongoing personal relationships and better and more consistent information. ‘Do not change contact person’ and ‘provide follow-up over time’ are suggestions mostly relevant for the above mentioned ‘Relationship’-theme. ‘Talk to each other’, ‘provide information about (planned) evaluations, treatments and support’, ‘convey the same messages’ and ‘include family in information and contact’ are suggestions relevant for the ‘Knowledge’-theme above. Furthermore, health professionals were asked to take service users’ vulnerability into consideration, and not to expect the person always to act perfectly in line with the expectations of the care providers. The following quote illustrates several of the themes. The young participant desired an ongoing personal relationship characterised by respect for her mental health problems and consistent information:

‘They could have given a bit more consideration to the fact that I struggle so much with anxiety. And I know from speaking with other people that suffer from anxiety like that, that people in the public sector ... [...]. It seems like they don't care, because if they did they wouldn’t move people without saying anything, for example. And they wouldn’t come with ten different messages and be so sure that what you did was wrong, because I can’t concentrate if I get ten different messages ... Then I just forget it, because I don’t have the energy for that.’ (P2 (T1)).

‘If they worked better together, how, in your opinion, would that affect your situation?’ (EB).

‘I think it would have been easier for me to meet them and look them in the eyes and admit when I am wrong, because I’m not perfect by any means, but you need to be for it to work.’ (P2).

‘Would you have felt more secure?’ (BS).

‘[...] I definitely would!’ (P2).

## Discussion

### Continuity of care as experienced by service users

The five continuums that were elicited through the analysis represent aspects of continuity that are essential from the perspectives of service users. The five themes were *Relationship, Timeliness, Mutuality, Choice and Knowledge*. Other recent studies performed within user-involved frameworks have defined the concept of continuity of care as having the following three dimensions: *Preconditions for continuity of care*, i.e. easy access to a range of services accompanied by high quality information and having the services that are needed to move forward, *Staff-related continuity of care*, i.e. good communication between staff and infrequent staff changes meaning that service users do not have to repeat their life histories to new staff, and flexible service responses, and *Care contacts,* i.e. not having to wait for services, being able to choose to avoid services and having support from peers, out of hours and through established crisis systems and day centres [14, 16, 45]. Both the present study and these earlier studies emphasise ongoing personal relationships, choice and flexibility as most essential dimensions of continuity of care as experienced by service users. Flexibility in terms of mutuality and choice emerge as even more important elements in service users’ experiences and definitions of continuity of care [9, 13, 14, 16, 45] than in the definitions that mainly represent the professional perspective [6].

### Ongoing personal relationships

The present results imply that improving personal continuity in mental health and welfare services should be a number one priority. Trusting relationships are central for recovery [46, 47], and recovery-oriented services are characterised by personal continuity in the partnership between the service user and his professional helper [48]. Many previous studies support ongoing personal relationships with the same carer as a paramount feature of continuity of care [16, 21, 24, 25, 27, 49–52]. Stable personal relationships form the basis for respect and mutuality. However, as in earlier studies, the participants in the present study experienced frequent discontinuities in their contacts with professional helpers [15, 17, 24, 53, 54]. Such breaks in interpersonal relationships were experienced as stressful, anxiety-provoking [15, 21, 25, 55] and left the service users feeling rejected, with less opportunity to contribute to their care plan [21, 22, 55, 56]. To people who have experienced frequent breaks in their relationships with significant others in the past, such interruptions in personal relationships in health and welfare services may be particularly devastating [57].

### Choice and mutuality

Service users in the present study called for mutuality and flexibility in their contact with professional helpers as well as the opportunity to choose the type and location of treatment and support. Further, they wished for opportunities to contact their professional helpers between scheduled appointments and across service levels. However, service users experienced the health and welfare services as a system with strict boundaries, within which they were expected to act in certain ways. The experienced rigidity and lack of mutuality encountered by service users gave rise to feelings of having to ‘fight’ the system, indifference and exhaustion. When offered a range of possibilities to choose from, service users chose services and staff they were familiar with, and who knew them and their personal history. They also opted for solutions that were practical within the context of their everyday life, taking family, social and geographical factors into consideration.

### Improvement in experiences of continuity of care is needed

In the present study, service users advised professionals to talk to each other, involve the service user and his next-of-kin and prioritise personal continuity and flexibility in the contact with service users. The participants felt that unnecessary deterioration of their mental health problems would be avoided if services had been more timely, and they would have felt more secure in their roles as service users if they were better informed about planned treatments and interventions. Earlier research suggests that improving the aspects of continuity of care that are central for service users may improve health and social outcomes [58, 59]. Recovery-oriented services are characterised by positive personal relationships and by being responsive to the needs and desires of the individual, and are focused on peoples’ right to make decisions about care and other aspects of their recovery [48, 60, 61]. Several recovery-oriented approaches in which collaborative partnerships, relational continuity and individual planning are central elements have shown beneficial results. For instance, crisis resolution teams and assertive community treatment teams [62] are service delivery models that aim for more active follow up and more flexibility in how team members meet the services users with severe and complex mental health or addiction problems. In the present study, the finding that service users experienced frequent breaks in their relationships with professional helpers and lack of mutuality and choice imply that health and social services need to facilitate organisational transformation at a structural level. Establishing collaborative partnerships between the service user and his professional helpers should be central aims in such a transformation. Further, system changes should include the implementation of communication tools allowing for communication with the service user and his next-of-kin, transfer of necessary information across units and service levels, and planning of interventions tailored to the person’s needs and preferences.

### Strengths and limitations of the study

We argue that the user-involved collaborative approach used in the present study increased the relevance and validity of its findings for the service user perspective [40]. The participation of service users in the development of research questions, piloting of the semi-structured interview guide, and analysis and interpretation of data assured validity and relevance of research questions asked and conclusions drawn. However, the study suffers from some limitations. Firstly, the process of engaging in an ongoing reflexive analysis is difficult [41]. Most of the researchers had professional backgrounds in mental health care and quality improvement, and although they were aware of how their basic assumptions and values could affect the questions asked and conclusions drawn, intersubjective elements may have influenced data collection and analysis. As in many earlier studies [63], theoretical definitions of continuity of care that mainly stem from the perspective of health professionals represented the starting point for the exploration of service users’ experiences of continuity of care in the present study. Despite the service user involvement in the study, this potential bias arising from the researchers’ professional background knowledge of existing definitions of continuity of care may have influenced which questions were asked and how data were interpreted. Although the study had two points of measurement, the longitudinal design was not fully taken advantage of. The exploration of intra-individual changes in attitudes and insights with regard to their experiences of continuity of care was limited, as only half of the participants had experiences with health care at the first interview, and as only a few questions about experiences with aspects of continuity of care were included in the semi-structured interview guide at the first time point. The finding that some participants reported overall satisfaction with care, in spite of experiences of discontinuities or insufficient information, may reflect that aspects of care other than continuity are important for service users’ satisfaction with services. Alternatively, their satisfaction with care could be a result of memory bias at the second interview, as the participants then had experienced improvement of their situation. Further, the satisfaction with care reported by participants could represent an eagerness to please the interviewers in the present study. Finally, the study was limited in that it included a small number of participants; the representativeness and transferability of findings may therefore be called into question. However, the inclusion of a range of common mental health problems and many services relevant to recovery may support the transferability of results. Further, we argue that by taking a comprehensive approach that includes a range of first-hand experiences of services relevant to recovery, the study reflects continuity of care as experienced by service users from their situation and context.

## Conclusions

Service users’ needs are complex and a range of mental health and welfare services are relevant to recovery. Five continuums defined in the present study represent essential dimensions of continuity of care as experienced by service users. Services that aim at being recovery oriented need to improve experiences of continuity of care by facilitating organisational transformation at a structural level. Mental health and welfare services should be organised in a way that allows for ongoing collaborative partnerships between service users and professionals. The valid evidence generated in the present user-involved collaborative study could therefore help inform policy makers, managers, professional helpers and service users in their common efforts to improve the organisation and integration of services and orienting them towards recovery. Further studies should focus on the planning, implementation and evaluation of organisational transformations developed based on evidence generated from the service user perspective.

## Additional files

Additional file 1: Guide for semi-structured individual interview. (DOC 48 kb)

Additional file 2: 32-item COnsolidated criteria for Reporting Qualitative research (COREQ) checklist completed for the study. (PDF 509 kb)

## Abbreviations

CMHC: Community mental health centre
GP: General practitioner
NAV: Norwegian labour and welfare administration
P1, P2, etc.: Participant 1, participant 2, etc.
T0: Time of first interview
T1: Time of the second interview; M: male; F: female
