# Supplementary material for: Continuity of care as experienced by mental health service users - a qualitative study
Source: BMC Health Serv Res. 2017 Nov 21;17:763. doi: 10.1186/s12913-017-2719-9 (PMC5698968; doi:10.1186/s12913-017-2719-9)
Supplement: Supplementary file 1 — Guide for semi-structured individual interview. (DOC 48 kb) [file 12913_2017_2719_MOESM1_ESM.doc]

**Additional file 1 Thematic guide for semi-structured interview***

| **Interview T0**  **At the start of treatment at a Community Mental Health Centre (CMHC)** |
| --- |
| **A) First encounter with health care services** |
| *First we will talk a bit about how things were at the time of your first contact with health care due to your problems….*  Do you remember the first time you needed help, and the first time you were in contact with health care services for these problems ? (or did others contact health care for you?)  -Who did you contact?  -What made you think that you needed to ask for help?  -Would contacting the helping services earlier been beneficial for you?  -What did you feel hindered you from starting treatment earlier?  What else have you previously tried to help yourself? |
| **B) The pathway to the CMHC this time** |
| Who referred you to the CMHC?  How did you experience being referred to the CMHC now?  -Can you describe the referral process?  -Did you get any information, did something happen?  How long did you have to wait for an appointment?  Can you tell me something about how waiting for the first appointment felt?  -In what way was it negative, and positive?  What did you do while you were waiting for an appointment?  -who did you spend time with?  -where were you?  -were any of these things helpful?  Is there anything someone can do for you in order to improve your experience of the waiting time?  Regarding the problems you struggle with, when would you ideally wished to come to the CMHC? |
| **C) The first encounter with the CMCH this time** |
| Is treatment at the CMHC something that you really desire yourself?  Can you say something about how your first encounter with the CMHC was?  -What did you feel as you were in the reception area, and in the waiting room?  What did you think of after the first talk with your health professional?  -Did you experience seeing the health professional as helpful or not?  -In what way was it helpful? Or not?  -Did you feel that you got the opportunity to share the stuff that was important to you with the health professional?  What else have the health professional or others at the CMHC helped you with? |
| **D) Aims for the treatment at the CMHC** |
| How do you wish the CMCH to help you?  -are there ways the CMHC can support you in ways other than therapy talks and medications?  Do you have personal goals for treatment at the CMHC? What are these goals then? |
| **Interview T1**  **Two years after baseline interview** |
| *Now it is approximately two years since we last talked. In the conversation back then you had just started your treatment at the CMCH. I would appreciate it a lot if we could talk a bit about your experience with using the CMCH, and possibly with other services you have used, and about how things have been for you. But first of all:*  Can you tell me how you are now?  What do you do nowadays?  -Who do you spend time with? Are there special places you like and where you spend time?  -Has life changed since the last time? Which important things have happened? |
| **E) Treatment at the CMHC** |
| *We are wondering if we could talk about how you are doing and what you have experienced in your contact with health care services since we last spoke:*  What types of services have you used since the interview two years ago?  *(keywords: therapist/health professional, out-patient clinic, hospital stays, day care unit, courses, social workers, Labour and Welfare Administration, general practitioner (GP), mental health community nurse, others?)*  We would appreciate it if you would tell us about your experiences with these various support services you have used …  -What have the staff at the CMHC/ in the support services done that has been of help?  -In what way has this been helpful?  *(keywords: thoughts, emotions, mastering, activity, hope…)*  -It would be nice if we could get to know whether there are things that have not been of help?  What do you think about the timing of the help you got, seen in the perspective of your problems?  -You had to wait for the first appointment at the CMHC, what do you think about this now in retrospect?  And what have you learned to do yourself in order to have a better situation since the last interview?  Are there others who have been of help *(for instance family, friends, colleagues, neighbours)?* |
| **F) Early support and later problems and functioning** |
| *In health care there is an ongoing discussion about the significance of early interventions.*  In your situation, how important is getting early support to you?  In your experience, do you feel that some of your early encounters with GP and CMHC have been significant with regard to how you are doing now?  -if so: can you tell us in what way?  *In the health care system, there are sometimes waiting times between the various treatments or appointments …*  Have you experienced waiting? When?  How did you experience being on a waiting list between the various health care services?  -What did you do to help yourself while waiting?  -Did anyone else do something that was helpful? |
| **G) More about experienced cooperation and continuity in health care and welfare services** |
| How –in your experience- did the cooperation between the CMHC, your general practitioner, and other contacts in health care or the Labour and Welfare Administration function?  Are there ways in which these parties could have collaborated better?’, ‘If these parties had worked better together, how, in your opinion, could this have affected your situation?  *(keywords: transitions between services, communication between services (letters, phone, other), planning, crisis interventions, information provided, agreement between professionals)*  Do you think their working better together would have meant something for your situation?  Could you now, in retrospect, have wanted other support interventions than those you did actually receive? If yes, what?  What kind of support do you ideally ask for from health care services, considering your current situation?  Is there something else that you feel health care service could do better?  *In case you still are using the CMCH:*  Have you had breaks in your contact there? For instance changed therapist or stopped seeing a health professional?  -What was the reason for this?  -Could you tell me about your experience with this?  *If you do no longer see someone at the CMHC:*  How did your contact there end/what was the reason?  Did this lead to any changes for you with regard to how you are doing or what you are doing? |
| **H) Further issues** |
| *We are now going to mention some specific treatments or interventions, and we are wondering whether*  *-You have been offered or have used any of these?*  *-If it has been of help or not?*  *-What you think of the intervention, could it be helpful?:*  -Individual planning  -phone contact  -Contact per e-mail  -Online information about mental health problems and their treatment  -Information about legal rights and possible interventions from the Labour and welfare administration  -Family meetings  -Home visits  -Electronic communication between health professionals at the CMHC, GP, and others  -Collaborative meetings between health professionals at the CMHC, GP, and others  *Please tell us whether these things have been helpful or not….*  Now we have talked about your experiences twice (two years ago and today), is there anything which in your view is different now compared to when we last spoke?  -If you had known what you know today, would you have done anything earlier in a different way (back when you started feeling bad)?  *The last time we spoke, you said that your aims for treatment and/or life was to….* (insert the participants’ personal aims and desires): ______________________________  -Have you achieved any of these? If yes, how?  What hopes and goals for the future do you now have?  Thank you very much! |

* Questions in sections B), F) (both at T0) and E), G) and H) (the latter three at T1) are central in the material analysed in the present sub-study

CMHC: Community Mental Health Centre

GP: General practitioner
